# Comparability of tumor-cytogenetics and -DNA-cytometry

**DOI:** 10.1186/s13039-015-0132-9

**Published:** 2015-04-18

**Authors:** Alfred Böcking

**Affiliations:** Institute of Pathology, City Hospital Düren, Roonstrasse 30, 52351 Düren, Germany

**Keywords:** Cancer cytogenetics, Diagnostic DNA-cytometry, Speciation-theory of carcinogenesis

## Abstract

Knowledge of the karyotypes of cancer cells is the theoretical underpinning of diagnostic/prognostic DNA-cytometry. Currently the translation is hampered by different terminologies of both fields. The following letter tries to close current gaps between the two fields by harmonizing their different terminologies.

## Why to publish this letter

Cytogenetics and DNA-cytometry both study the karyotypic basis of the malignant transformation of cells. Their results are however rarely compared with each other, although both could profit from such comparisons. On the occasion of the excellent article by Bloomfield et al. in Mol Cytogen 7:71 (2014) this letter tries to harmonize the terminology of both fields to advance mutual understanding and thus the impact of both fields.

With great interest and appreciation I have read the excellent article: “Karyotypic evolutions of cancer species in rats during the long latent periods after injection of nitrosurea” by Bloomfield et al. in Molecular Cytogenetics, 7:71 (2014).

As a cytopathologist, dedicated to diagnostic and prognostic DNA-image-cytometry for 30 years, I have been interested to find out whether the „karyotypic-“ or speciation-theory” of the malignant transformation of human cells by Bloomfield et al. is consistent with my observations on the occurrence and development of DNA-aneuploidy in different types of non-neoplastic, preneoplastic and malignant human lesions. The result is: Yes, if we make the following translations:

## Clonal-/DNA-stemline aneuploidy = malignancy

What Bloomfield et al. call “**clonal aneuploidy**” corresponds to **“stemline aneuploidy”** in DNA-cytometry. It is 100% specific for malignancy. We could prove that if DNA-aneuploidy is present in lesions that are not (yet) morphologically diagnosed as definitely malignant by pathologists (atypical hyperplasias, dysplasias), these lesions will develop histologically detectable malignancy within a few months to years [1-3]. We thus call these lesions: “prospectively malignant” [4-6]. The **clonal aneuploidy/DNA-stemline-aneuploidy** described by Bloomfield et al. defines in our experience any tissue-lesion as malignant or obligatory premalignant. The Positive Predictive Value, PPV, of clonal aneuploidy to develop histologically proven malignancy, if untreated, is 100%. Such lesions have to be treated immediately by removal.

## Non clonal-/DNA-single-cell aneuploidy = possible malignancy

**Figure 1:**
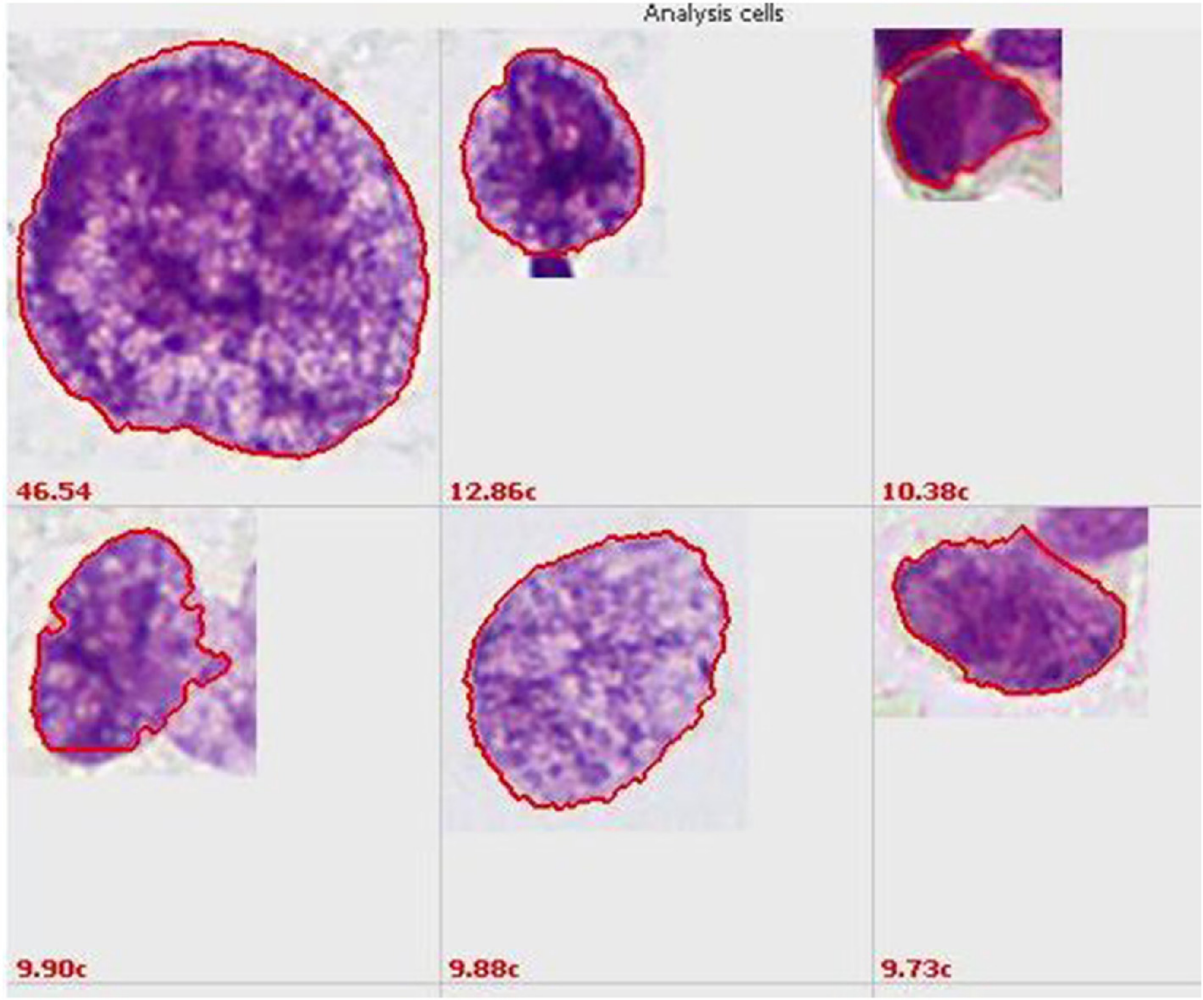
Rare nuclei with abnormally high DNA-contents in c, correspondig to non-clonal aneuploidy. 1c = DNA-content of one haploid chromosomal set. From a bile-duct brushing with in-situ cancer. Specifically stained for DNA according to Feulgen.

What Bloomfield et al. call “**non clonal aneuploidy**” results in “**single-cell aneuploidy**” in DNA-image-cytometry. We call these abnormal DNA-values of single cells “rare events” (Figure 1). The detection of non clonal aneuploidy in atypical hyperplasias or dysplasias is associated with a lower PPV in subsequent months as compared with DNA-stemline-aneuploidy (e.g. 41,2% as compared to 76,3% for Pap-smears: Grote et al., [3]). As this PPV is too high to be left for surveillance only, we also recommend therapy of the respective lesions or at least tight follow-up with repeated biopsies.

## Number of abnormal clones/DNA-stemlines = grades of malignancy

**Number and abnormality of cytogenetic clonal variants** in a given cancer are equivalent of their genetic flexibility. Such clones have their cytometric equivalent in the **number and abnormality of DNA-stemlines.** Both parameters correlate well with the malignant potential of an individual cancer. They therefore are suited as prognostic markers for grading the malignancy of cancers [7-10].

**DNA-stemlines,** with **clonal aneuploidy,** are equally well identified by image- and flow-cytometry Motherby et al. [1]). N**on clonal aneuploidy** of **single cells** with abnormally high nuclear DNA-contents, is however only detected with sufficient sensitivity by image-cytometry (Figure 1).

## From hyperplasia to malignancy

Hyperplasias and dysplasias do not generally, or a priori contain non clonal or **single cell aneuploidy**, as they can start as mere diploid hyperproliferations. Without any type of aneuploidy they do not yet bear a clinically relevant risk to become malignant. Yet, they are at risk to develop aneuploidy. As long, as we do not detect any type of aneuploidy in these lesions, we do not call them “prospectively malignant” or “premalignant”. But, the risk of a hyperplasia to become malignant is slightly increased by the increased rate of proliferation, which is associated with an increased risk to develop “**non clonal aneuploidy**”. The risk of a hyperplasia with “**non clonal aneuploidy**” (“**single cell aneuploidy**”) to develop histologically manifest cancer is already so high as to justify clinical intervention [2,3].

Thus hyperproliferation in hyperplasias or dysplasias, even without known carcinogens, are at risk to develop non-clonal and subsequent **clonal aneuploidies**. As soon as this has occurred, the lesion has to be considered as definitely malignant (= cancer, if it is derived from epithelial cells).

Moreover, carcinogens are able to induce **non clonal aneuploidy** (Figure 1) even without the occurrence of hyperplasias, as for example asbestos-induced mesothelioma, radiation-induced lung cancer and tobacco-induced oral and cervical cancer.

Best regards

Alfred Böcking

## Abbreviations

PPV: Positive predictive value
